# Parenting and oral health in an inner-city environment: a qualitative pilot study

**DOI:** 10.1186/s12903-018-0584-5

**Published:** 2018-10-20

**Authors:** Shalini Nayee, Charlotte Klass, Gail Findlay, Jennifer E. Gallagher

**Affiliations:** 1 King’s College London Dental Institute, Population and Patient Health Division, Denmark Hill, London, SE5 9RS UK; 2 Institute for Health and Human Development, University of East London Stratford Campus, Water Lane, London, E12 4LZ UK

**Keywords:** Oral health promotion, Parenting, Oral health, Access to dental care

## Abstract

**Background:**

Preventable oral diseases such as dental caries remain common in the United Kingdom. Clustering of poor health is observed within deprived communities, such as inner-city areas, where elevated levels of dental need are associated with lower uptake of dental care. Successful oral health promotion (OHP) initiatives are contingent upon effective community engagement. The aim of this pilot study was to engage with families with young children to explore community views on oral health and dental care and thus tailor OHP initiatives more effectively to their needs.

**Methods:**

Qualitative research, involving individual interviews and triad focus groups with parents/caregivers, was conducted in a south London inner-city community as part of a ‘Well London’ programme initiative.

**Results:**

Seventeen parents/caregivers participated in this pilot study. Parents/caregivers described a spectrum of oral health behaviours based on their social history, past dental experiences and cultural influences. All parents described a clear desire to create healthy lives for their children; however, two broad groups were apparent, termed ‘Oral Health Prioritisers’ and ‘Oral Health Non-prioritisers’. The former reported regularly accessing dental care for their children, believing that oral health contributes to systemic health. Non-prioritisers, however, preferentially used key services considered most beneficial to their child’s wellbeing. Dental services were considered a low priority for this group, where oral health was synonymous with absence of pain. Participants in both groups favoured OHP initiatives involving a range of health and social care services, with schools at the epicentre of programmes. First-time parents were proposed as an important group requiring support in future OHP initiatives with evidence suggesting that first-born children may have delayed presentation to a dentist.

**Conclusions:**

The findings suggest that this inner-city community may contain sub-groups with contrasting perspectives on oral health and oral health behaviours; nevertheless, there was support for a systems approach to oral health promotion initiatives involving a range of health and social care services, including a critcal role for schools, and actively connecting with first-time parents. The findings provide the basis for further research.

**Electronic supplementary material:**

The online version of this article (10.1186/s12903-018-0584-5) contains supplementary material, which is available to authorized users.

## Background

Parents living in deprived, inner city environments face an array of challenges to establish and maintain healthy lifestyles for their families. Common risk factors for chronic oral and systemic disease, such as poor diet, obesity and lack of exercise, disproportionately impact upon deprived families [1], due to clustering of multiple risk factors within such communities [2]. These effects are further exacerbated within inner city areas due to the convergence of multiple factors including mobile populations, weak social networks [3], financial insecurity and lack of engagement with preventive health services [4], resulting in worse systemic and oral health compared with more affluent communities.

Lambeth, a densely populated inner London borough with high levels of deprivation, faces a spectrum of dental and general health challenges, exemplified by lower life expectancy for its residents than in London and England overall [5]. Oral disease is common, with approximately 10% of 3-year old [6], and 24% of 5-year old children [7], found to have at least one untreated carious tooth. Nevertheless the objective ‘need’ for oral care does not translate to perceived need for oral care amongst its residents, with dental attendance rates persistently lower than London and national averages, particularly amongst children [8]. Recent data show that 41% of Lambeth children did not access any NHS dental services over a two-year period [8], with this figure, encompassing all parts of Lambeth, masking substantially worse access rates within northern localities of the borough. Amongst adult residents the situation appears worse with 49% of adults not accessing any NHS dental services over a two-year period [8], suggesting a prevailing culture of irregular dental attendance, albeit that some may possibly choose to access services privately. The dichotomy between high levels of dental disease but persistently low uptake of dental services suggests there is a poor ‘fit’ between the community and local dental services [9].

Low uptake of primary care dental services leads to more advanced presentation of dental disease, with provisional data showing that dental caries is the most common primary diagnosis amongst young children in England requiring admission to hospital [10]. Moreover, children from deprived communities are over-represented in such admissions, such that they are twice as likely to require hospital admission for dental care as children from the most affluent communities [11].

Increased uptake of dental services represents a fundamental step in improving oral health within this community, and similar communities, not only giving local residents exposure to evidence-based preventive care and advice [12], but also contributing to improved oral health awareness, which can be sustained in the community for future generations. Recent oral health statistics for 3-year old children showing high caries experience amongst this cohort [6], also highlight the critical importance of early access to dental care, with childhood oral disease an important predictor of future oral disease.

Oral health and uptake of dental services can be improved significantly through appropriate oral health promotion (OHP) initiatives [13]. Moreover, targeting of these initiatives towards families with young children provides a pragmatic way to improve both current and future oral health outcomes, with this child-centric approach mirroring recent recommendations for transforming public health in London [14]. OHP initiatives are deemed successful if they provide sustained improvement in health outcomes, with the onus on local communities to facilitate this effort. Ensuring that OHP initiatives can be sustained by local communities requires detailed community consultation during planning and development, as parental knowledge, attitudes and beliefs may differ widely from those of dentists and service planners/commissioners [15]. Community engagement is also critical for improving health equity, acting to improve social cohesion, foster mutual responsibility and empower the local community [1].

The aim of this qualitative study was to investigate how parents/caregivers with young children perceive oral health; to understand the key factors and beliefs that have shaped these perceptions of oral health; and to understand how these perceptions affect oral health behaviours, including uptake of dental care. These findings will be used to provide insight into how OHP initiatives can be formulated to improve oral health within this community, and similar communities.

This study was nested within the wider public health ‘Well London’ programme which involves community engagement and is based on a development framework for communities and local organisations to work together to improve health, wellbeing and reduce inequalities [16]. A three-year Well London (Phase 2) initiative was focussed in the neighbourhood where the parents/care giver respondents in this study were residents [17], to inform specific dental aspects of the programme.

## Methods

This study involved a dual method qualitative approach, encompassing individual semi-structured interviews and triad focus groups of up to three participants [18], to consult with parents/caregivers of young children. Hereafter, parents/caregivers will be referred to as parents to enhance the flow of the paper.

Parents (aged 18 years and over) living in a North Lambeth estate, or its proximity, were invited to participate in this pilot study through recruitment via local community events, children’s centres and schools. Research ethics approval was sought and obtained from King’s College London Research Ethics Committee (BDM/13/14-29); this included consent to publish participants’ data.

Verbal and written informed consent was obtained from all parents agreeing to take part in the Additional file 1. Parents were also invited to complete an information sheet, providing the researchers with basic demographic information. Participants were assigned to individual interviews or triad focus groups based on their personal preference. Triad focus groups provided parents with an opportunity to interact with one another other and explore novel themes that emerged from these interactions, but on a sufficiently small scale that more personal or sensitive topics could be discussed. Individual interviews provided parents with a preference for one-to-one conversations with an opportunity to share their views with researchers.

Interviews and focus groups were conducted by two female researchers (SN and CK). Interview/focus group venues were selected based on ease for informants, with use of local community centres and children’s centres. Topics for discussion during interviews/focus group were outlined in a pre-determined topic guide, informed by the Additional file 2. These included to: perceptions of oral health; oral health knowledge and behaviours; barriers to oral care; and oral health promotion initiatives to improve oral health and increase uptake of dental care.

Focus groups lasted up to 35 min and interviews from 30 to 65 min. Interviews/focus groups were arranged until saturation was reached: in total 17 parents were consulted in this study, through a combination of eight individual interviews and four triad interviews.

### Data analysis

All data were transcribed and analysed using the ‘framework’ method by Ritchie and Lewis, 2003 [18]. This iterative process entailed listening to audio recordings and reading transcripts obtained from the first six interviews/focus groups. These initial interviews/focus groups were coded, from which initial categories were identified by three researchers (SN, CK and JEG). Categories and themes were used to construct an initial analytical framework, to which relevant codified sections of interviews/focus groups were added by a single researcher (SN). Subsequent interviews/focus groups were analysed, and the analytical framework modified to reflect additional emergent themes, followed by creation of a summary framework matrix. Finally, data interpretation was performed to distil the content and meaning of the data into underlying theoretical principles, with construction of typologies to broadly define and explain the differing views within the sample population.

## Results

Approximately 80 parents were provided with a summary of the study, half of whom declined to receive any further information, most commonly citing lack of time due to work commitments. It was also apparent that a small sub-group of parents had challenging personal circumstances that rendered their lives too chaotic to consider participating in this study. Amongst the 40 parents who did voice initial interest in participation, approximately 20 parents were lost due to the requirement for parents to spend at least 24 h considering their potential participation before being contacted again by researchers, in line with ethics committee stipulations. Eight parents participated in individual interviews and nine in triad interviews/small focus groups.

### Participant demographics

Interviews/focus groups were performed until a representative sample was obtained, and saturation was reached with respect to the views and ideas expressed by informants. In total 17 individuals participated in this research, comprising 15 mothers, one father and one grandmother. Available demographic information for all informants is summarised in Table 1. Approximately 50% of the study informants were first generation migrant parents who had not been raised in the UK. In presenting the findings, parents will be denoted with the abbreviation ‘P’ and the grandparent with ‘GP’, females as [F] and the male informant as [M].

**Table 1:** Demographic details of informants participating in individual interviews and triad focus groups

Gender	Number
Females	16
Males	1
Age group	
25–34	9
35–44	6
45–54	1
55–64	1
Relationship to children	
Parent	16
Grandparent	1
Ethnicity	
White British	3
Black (Unspecified)	6
Black (African)	5
Black (British)	1
North African	1
South American	1
Residence	
Within the estate	6
In close proximity to estate	11
Number of children	
1	5
2	9
3	1
4	1
5	1

### A community with divergent perspectives of oral health

**Fig. 1:**
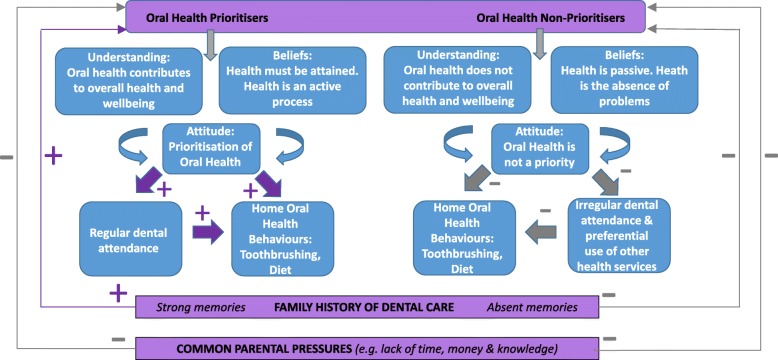
Parental typology: understanding, attitudes, beliefs and implications

In common with many inner-city environments, the population of this South London community is characterised by the diversity of its residents, with respect to ethnicity, socio-economic status and cultural background. This diversity is reflected in the study sample and the spectrum of attitudes, views and beliefs that were encountered throughout this study. At either end of this spectrum were two distinct views of oral health and patterns of oral health behaviours (Fig. 1). ‘Oral Health Prioritisers’ were motivated by the underlying belief that oral health forms an integral component of a child’s overall health and wellbeing, attained through specific habits, routines and behaviours, as summarised by one mother:

*[It’s] The food you eat, the drinks you drink and the way you brush your teeth* (P12[F], 9.422)

In contrast, ‘Oral Health Non-prioritisers’ considered oral health to be unrelated to a child’s overall health and wellbeing, believing oral health to be synonymous with lack of symptoms, notably pain. These views were exemplified by one respondent who had lived in the community for 10 years and had not taken their children to a dentist:

*I just think the dentist is a bit … [low priority] … to be honest with you I don’t know where the dentist is* (P2[M], 2.072)

Oral Health Prioritisers were often critical of Oral Health Non-prioritisers, attributing their behaviour to lack of care for their children, or even laziness. One grandmother suggested that dental anxiety may contribute to the irregular dental attendance of Oral Health Non-prioritisers, although she did not feel this excused their behaviour:

*It’s probably not a case of not wanting to take them, maybe the parents were scared of the dentist themselves…or they were working and could not take them to the dentist, but I am sure there could have been a way that parents could take time out to take the child to the dentist (GP[F], 8.290)*

### Selective engagement with health services

Parental perceptions of oral health made an important contribution to use and knowledge of local dental services, with contrasting dental attendance patterns between Oral Health Non-prioritisers and Oral Health Prioritisers. The effects of lower overall health literacy and health awareness amongst Oral Health Non-prioritisers, compared with Oral Health Prioritisers, were evident with respect to dental attendance. There was lack of awareness of professional preventive dental care amongst Oral Health Non-prioritisers, with some parents never having been to the dentist, or only having visited the dentist for the first time during adulthood. There was a common assumption that dental services in London are predominantly private, acting to reinforce the belief that dental care is a non-essential health service, which should only be accessed for specific problems. Oral Health Non-prioritisers described dental visits in the context of being ‘forced’ to go and discussed costs of dental care in terms of ‘fear’:

*...most of them are private. So… you are scared* (P2[M], 2.102)

One mother, who had never been to the dentist, considered private care to be a major obstacle to seeking dental care, even when in pain:

*…if I don’t have enough money to go to the private dentists, that means even if I’m in pain I prefer taking paracetamol* (P16[F], 12.309)

Some Oral Health Non-prioritisers also cited the practicalities of accessing dental care as a substantial barrier to accessing dental care. One mother, aware of her family’s entitlement to free dental care and of the notion of attending the dentist for a ‘check-up’, had only taken one of her five children to the dentist:

*It’s important to go to the check-up, but I never go… I’m busy that’s why.* (P11[F], 7.258-7.260)

In theory, these barriers (with the exception of cost) could be applicable to all preventive health services within the community. Nevertheless, all Oral Health Non-prioritisers in this study described voluntarily engaging with community baby clinics and children’s centres for preventive care and advice. It was apparent that Oral Health Non-prioritisers restricted their engagement to a limited number of key health services they considered most beneficial to their children’s health. The perceived irrelevance of oral health to overall health/wellbeing rendered dental care a low priority service.

The importance of underlying belief systems is underscored by the fact that the majority of Oral Health Prioritisers interviewed described a range of barriers to accessing dental care. These included: concern about the costs of NHS care; being advised that routine NHS care was only available privately; lack of space in dental practices for children’s buggies; suboptimal communication from members of the dental team; adverse treatment outcomes; and difficulty obtaining appointments during school holidays. Despite these barriers to care, Oral Health Prioritisers described patterns of regular dental attendance for their families, suggesting that beliefs regarding the importance of oral health were a critical determinant of dental attendance.

### The power of history

Investigation of parents’ perceptions of oral health and oral health behaviours highlighted the critical influence of past experiences. It was apparent that parents considered their own childhood experiences to be a key driver of their current behaviours. Many Oral Health Prioritisers attributed their good oral health behaviours to repeating and copying the actions of their own parents, as described by one mother:

*... every six months we went... all of us went to the dentist and we always brushed our teeth morning and night and that's the way it was... So that's what I do with my daughter. (P14*[F], *L10.116 - 10.118)*

Oral Health Prioritisers raised in the UK recounted enjoying dental appointments during childhood, recalling the excitement of leaving school to visit the dentist, although there were also negative memories such as having treatment under ‘gas and air’ which inevitably meant that they would have had teeth removed under general anaesthetic or sedation. Some Oral Health Prioritiser migrant parents reported adapting their childhood routines to their new environment and resources, as described by one mother raised in West Africa. She had implemented a strict routine of regular dental visits and twice-daily supervised toothbrushing regime for her children, attributing her current fastidiousness to her own childhood experiences:

*...I encourage [children] to clean their teeth, which sometimes is not easy...I said to [son], you are so lucky, you’ve got toothbrush, you’ve got toothpaste...I can remember... we go overnight, after they’ve cooked with the wood, in the morning the ash is cold and that’s what they will give you to go and scrub your teeth, and you scrub your teeth until they are squeaky clean. (P7*[F]*, L4.014-4.018)*

Repetition and copying of childhood experiences was also observed amongst Oral Health Non-prioritisers, with none of these parents reporting a history of regular dental attendance during their own childhoods. Indeed, amongst many parents there was a complete absence of childhood memories of home oral care or dental attendance, whether positive or negative. Lack of positive oral health behaviours during their own childhoods reassured some Oral Health Non-prioritisers that their current behaviours would not be detrimental to their child’s health. Other parents felt that changing some elements of their own childhood experiences would be sufficient. One mother with a childhood history of irregular dental attendance summarised her feelings by saying:

*... I guess, to be honest, I don’t see [visiting the dentist] as a priority now. As long as I know that I’m doing what needs to be done in terms of brushing ... Yeah, it’s not really much of a priority (P15*[F], *L11.097)*

### The need for OHP initiatives

All informants, whether Oral Health Prioritisers or Oral Health Non-prioritisers, demonstrated limitations in their oral health knowledge. In fact, an inverse relationship between parents’ self-perceived oral health knowledge and objective knowledge was evident. For example, many parents described limitations in their knowledge about toothbrushing, yet provided detailed descriptions of their family’s twice-daily brushing regimens. In contrast, the majority of parents reported confidence in their dietary knowledge yet probing of dietary practices revealed large gaps in knowledge, with descriptions consistent with children consuming diets with high cariogenic potential. Many parents found it challenging to restrict sugar, making reference to others, particularly grandparents and family friends, providing sweets; teachers using sweets to reward pupils; or children simply having a ‘sweet-tooth’. Moreover, some parents demonstrated confusion about the role of sugar, as described by one parent:

*Well sugar is not good, but kids need sugar to grow* (P2[M], L2.055)

Consumption of fruit juices was prevalent amongst the majority of families in this study, with fruit juices perceived as preferable to carbonated drinks, as described by one Oral Health Prioritiser mother:

*What they need to do is open more juice bar things or even in schools, have a little juice bar, say try this, try this, you can give out samples, let the kids decide what they like.* (P1[F], L1.206)

Discussion about dental visits also revealed that although the children of some informants had no caries experience, others across the spectrum of behaviours, knowledge and beliefs, from Oral Health Prioritisers to Oral Health Non-prioritisers, made reference to their children requiring fillings or other operative dental treatment.

### The importance of connecting – Making a difference

All participating parents acknowledged that OHP initiatives would be of most benefit if they adopted a systems approach, encompassing a range of health and social care services. The power of such an approach (both formal and informal) was apparent through the stories of parents who had transitioned from being an Oral Health Non-prioritiser to an Oral Health Prioritiser. For example, one migrant mother was hospitalised when she initially arrived in the UK, bringing her into contact with social workers. Their assistance facilitated engagement with primary care health services, and some two decades later, she attributed her health awareness and positive health behaviours to this early support. Another mother with a history of symptomatic dental attendance recalled a passing comment from her General Medical Practitioners (GMP) regarding registering her two-year old daughter with the dentist:

*I think the GP told me to… and they registered her, and they said we need to go to have the first appointment so that’s why I went. (P10*[F], *6.122)*

After this initial appointment with the dentist, the family developed a routine of regular dental appointments, demonstrating the utility of informal OHP for increasing uptake of dental care and fostering positive oral health behaviours.

### Lack of oral health literacy amongst first-time parents

Whilst parents in the community had diverse views on specific OHP initiatives, there was clear sentiment on who should be targeted by OHP initiatives, namely first-time parents. Many informants described the overwhelming nature of being a first-time parent, with lack of information forcing parents to rely on instincts and ‘common-sense’. The majority were unsure about the correct age to start brushing their children’s teeth, or when the first dental visit should be made. As a consequence, parents reported that first-born children often had their first dental visit much later than younger siblings.

Parents felt that dietary advice was more readily available for first-time parents, with health visitors and children’s centres being an important source of information. Parents with minimal contact with dentists were often reliant on health visitors and GMPs for oral health information, although the extent and standard of information provided was variable, as described by one young mother:

... *I don’t think there was a discussion [with the health visitor] about the dentist...because he was quite young then...in my mind anyway, you don’t really think he needs to start going to the dentist when he’s a baby, so yeah, that was it, it was more about brushing and how many times to brush and that kind of thing* (P15, L11.006)

### How to support first-time parents

Preference for a systems approach encompassing GMPs, health visitors, children’s centres, baby clinics and pharmacies was common amongst parents. Notably, there was little mention of desire for increased support from dentists themselves. This was particularly evident amongst parents who had little engagement with dental professionals and services. Many parents welcomed the idea of increased written information in the form of posters and leaflets, with distribution in antenatal classes, baby clinics/immunisation centres and children’s centres. Parents also favoured OHP delivered in group settings in baby clinics and children’s centres, enabling them to establish support networks with other parents, and perceiving this approach to be less intimidating than information delivered via health visitors in their own homes.

### A critical role for schools

Parents suggested a wide range of OHP initiatives they felt would benefit their community, and similar communities, and a summary of views is provided in Table 2. Participants were keen for schools to be at the epicentre of community-wide OHP initiatives, with readily available access to all school-age children, and minimal disruption for busy parents. School dental screening was popular, and considered by many the most equitable method of increasing uptake of dental care, for others, as summarised by one grandmother on behalf of the community:

*Because some children just didn’t go to the dentist. And somebody needed to help those children whose mums couldn’t be bothered to get off their bottoms and take their child to the dentist (GP1, 8.288)*

Initiatives to increase exposure to fluoride via toothbrushing and fluoride varnish schemes were broadly supported, although there were contrasting views on the practicalities of toothbrushing schemes, particularly in relation to infection control:

*...because they are taught to be so independent in school it freaks me out a bit that my son would pick up someone else’s toothbrush and use it* (P6 [F], L3.102)

*It is a good idea. Everybody’s got their own toothbrushes … let’s say they’re doing PE…everybody’s got their PE kit, everybody knows their PE kit. So it’s the same, everybody’s got their toothbrush, everybody knows their toothbrush, so that is a good idea* (P7[F], L4.118)

Overall, many parents felt that additional support from schools was critical to supplement their personal efforts to implement positive oral and general health behaviours, due to the considerable pressures of family life, common in this and similar communities, as described by one mother:

*...we have got enough as mothers to, you know, deal with at home. And you know you can be mum, you can be dad, you know, you are the referee, so it’s nice that, you know, school do back up what you are saying at home* (P6[F], L3.043)

**Table 2:** Summary of parental views on different OHP initiatives

OHP initiative	Level of parental support High = Majority support Medium = Support for initiative, with caveats Low = Lack of overall support	Summary of parental views
Improving oral health		
Fluoride Varnish Schemes	High	• Lack of awareness of fluoride varnish amongst the majority of parents • Most parents supported idea after initiative summarised • Parents keen for implementation via schools rather than in the community.
School-based toothbrushing clubs	Medium	• Most parents felt initiative would help busy parents • Concerns about infection control amongst parents with highest levels of health awareness • Some concerns about potential for disruption on lessons. • Many parents reported more problems with toothbrushing at weekends (due to loss of normal routine) than during school-days
School dental education sessions	Medium	• Likely to have an impact as children more receptive to information delivered by external speakers (e.g. dentists, dental nurses/hygienists, dental students) than their parents • Sessions must be interactive to engage pupils • Sessions will only have a transient effect if parents are not sufficiently involved
Dental education sessions for parents	Medium	• Parents are always keen for information that may benefit that child’s health and wellbeing • Lack of time to attend during working-hours • Community drop-in sessions suggested, but may not attract many parents
Healthy eating initiatives in schools	High	• Concern about unhealthy school dinners and use of sweets/chocolates to reward pupils • Initiative does not target fast-food outlets in close proximity to local schools
Increasing uptake of dental care		
School-based dental screening	High	• The only method of ensuring all children are seen by a dentist • UK-raised parents who recalled dental screening in their childhoods very supportive of reintroduction
Distribution of dental passports	High	• Simple but effective method, as frames dental visits in a positive way • Distribution through schools is easiest method
Text message reminders	High	• Would encourage preventive dental care • Should come via schools, as many already operate a text messaging service

## Discussion

This pilot study has identified two distinct groups of parents within this inner-city community. One group (termed Oral Health Prioritisers) considered oral health to be attainable through a combination of diet, oral hygiene and regular dental attendance, whilst the other (termed Oral Health Non-prioritisers) considered oral health to be synonymous with an absence of symptoms and reported irregular patterns of dental attendance.

Lack of familiarity with preventive dental care has previously been reported amongst migrant mothers in Australia [19], and this present study reflects this finding to an extent. Some first-generation migrant parents participating in focus groups were surprised to learn they could take their children to a dentist for preventive care as well as interventive treatment. Similarly amongst some non-migrant parents there was no awareness of professionally delivered preventive care. On the other hand, some first-generation migrant parents reported having implemented strict oral care regimens for their children, with respect to diet, oral hygiene and dental attendance. Clearly, factors beyond migration patterns have shaped the two distinct perspectives of oral health observed in this study.

Within this community, the decisive factor appeared to be whether parents believed oral health to be a component of their child’s overall health and wellbeing. All parents described a clear desire to create healthy lives for their children, and for Oral Health Prioritisers’ oral health was perceived to be a component of systemic health. In contrast, Oral Health Non-prioritisers considered oral health to be entirely distinct from overall health and wellbeing and accorded little priority to oral health. Oral Health Non-prioritisers were willing to engage with preventive services relating to systemic health (via children’s centres and baby clinics) but rarely, if ever, reported engaged with local dental services.

The beliefs and actions of these two groups of parents were reportedly shaped by past events, especially childhood experiences. It has been observed that negative experiences of dental care during childhood may manifest during adulthood, through parents delaying dental treatment for themselves and their children [20]. In our study, a striking absence of any memories relating to childhood dental visits was observed amongst Oral Health Non-prioritisers. In contrast, Oral Health Prioritisers had strong memories of childhood dental appointments, and whilst primarily positive, some were negative. Crucially, all parents reporting a history of regular dental attendance during childhood were Oral Health Prioritisers. This finding underlines the importance of parenting skills and encouraging early access to dental care for families, as childhood dental attendance patterns strongly shape attendance patterns during adulthood [21].

Both groups of parents identified additional barriers to dental attendance that have previously been documented in the literature [22]. These included time, cost, dental anxiety, concerns about availability of NHS care and poor communication from the dental team. Notably, language was not identified as a particular barrier to accessing dental care, although this may reflect the study methodology, as some parents with weaker English language skills were reluctant to participate in focus groups/interviews. This study has also highlighted the importance of community consultation for understanding local barriers, which may otherwise remain undiscovered. Within this community, there were specific concerns regarding the visibility of local practices, their size and the difficulties of taking pushchairs/buggies into these facilities.

The potential for some parents to ‘transition’ from being a Non-prioritiser to an Oral Health Prioritiser is an interesting finding in this pilot. Supporting a positive transition is critical to increase uptake of dental care and improve oral health and requires further exploration. Equally important, is the observation that no parents reported the converse; however, given the small numbers involved, caution should be exercised. The discovery of two distinct groups of parents within this community suggests that whilst it may be possible to direct some initiatives towards the whole community, most likely through schools and the wider health and social care systems, tailored initiatives will also be needed for those who don’t prioritise dental care. Particularly as OHP initiatives in dental practices will have little scope to reach Oral Health Non-prioritisers who rarely access dental services; first-born children may have delayed presentation to the dentist compared with younger siblings; and, parents will not have access to school initiatives until their firstborn is around 5 years of age.

In this context, the potential benefits of a systems approach are suggested by these findings, harnessing the influence of children’s centres, schools, GMPs and other health professionals to signpost dental services and embed OHP initiatives within the community. This approach is in accordance with current national and international health promotion programmes. Positive early experience is recognised as vital to ensure children are ready to learn, ready for school and have good life chances [23], and it is vitally important to make an overt link to dental services in support of socially deprived families.

This group of parents considered schools as significant resource for supporting oral health, an approach for which there is much support. The World Health Organisation (WHO) has identified schools as a critical setting for health promotion, enabling targeting of not only students, but the wider community of staff and parents [24]. Their Global School Health Initiative promotes the concept of ‘Health Promoting Schools’, with oral health promotion a key aspect of this initiative due to the high global burden of preventable dental disease, and shared risk factors for oral and systemic disease [24]. Moreover, national programmes within the UK such as ChildSmile in Scotland [25], and Designed to Smile in Wales [26], have demonstrated the feasibility and efficacy of OHP programmes that combine community initiatives with nursery and school-based programmes. This systems approach may have the added benefit of protecting individual services from excessive additional workloads, as areas of deprivation often exhibit an inverse care law, where there is a mismatch between the high needs for healthcare and supply of healthcare [27]. Residents in the most deprived communities are more likely to have multiple co-morbidities and psychosocial problems [27], resulting in complex health needs that may be best managed with multidisciplinary input.

One final consideration with respect to designing OHP initiatives is the possibility of additional sub-groups within this community that were not reached within this study. A plausible extension of the typologies defined in this study would be a third group of parents who appear to neglect both oral and systemic health, so-called ‘Health Non-prioritisers’. The current findings should therefore be tested on larger samples across a range of inner-city culturally diverse settings to gain deeper understanding of parents’ views and supportive action. The methods used in this study may have created an inherent bias, whereby parents who pay little heed to health and wellbeing do not use services where study recruitment was undertaken, have little interest in engaging with health researchers or participating in community consultation, or, have lives that are too chaotic to find time to participate in an interview or focus group when approached through schools. This group of parents undoubtedly represents those most in need of support via OHP initiatives and it would seem likely that a systems approach could assist with reaching this group of families.

### Limitations and strengths of study

This study presents the findings of interviews and focus groups with 17 parents in an inner city South London community. Whilst this is a relatively small sample size, it exceeds the acceptable size for hard to reach groups [28], and robust qualitative research [29]. Whilst there were informants from the main ethnic groups on the estate, younger parents, aged 16–24 years, did not participate despite efforts to recruit these individuals; younger parents may have specific views and beliefs that are not represented in this study. The lack of male participants is also acknowledged, although it is noted that the vast majority of primary caregivers encountered by the researchers in this community were female. Whilst parents with limited English language skills were invited to participate in this study, it is accepted that parents with the most limited English language skills may also have been deterred from participating in this research due to its qualitative nature. Future research should enable interviews to be undertaken in their mother tongue or via a translator, either of which has resource implications.

Research exploring oral health in inner city communities tends to focus on specific ethnic or cultural groups; this study provides insight on review of the attitudes and beliefs shaping participant’s oral health behaviours, and as such, many of the findings may be applicable to other diverse inner-city communities. Whilst these results provide insight to the perceptions and beliefs of those interviewed, further research is required to test and develop these findings to provide more generalisable insights. Future studies, exploring such issues, should gain ethical consent to interview parents when they are approached, and found to be willing and interested to participate, rather than arranging appointments to do so. These parents should of course be able to withdraw their data within an agreed time frame if they change their minds, without giving a reason.

## Conclusion

In conclusion, the evidence from this pilot study suggests that sub-groups within this inner-city area may place different emphases on oral health. None-the-less, participating parents favour a systems approach to OHP initiatives, involving a range of health and social care services, including a critcal role for schools, and actively connecting with first-time parents. These approaches may assist in reaching families who rarely engage with dental services and enable specific OHP initiatives to be delivered within this community. The findings provide the basis for further research.

## Additional files

Additional file 1: Appendix 1: Topic guide for focus groups and individual interviews. (DOCX 138 kb)

Additional file 2: Appendix 2: Questionnaire for participant demographic data (‘Participant information Sheet’). (DOCX 107 kb)
