# Supplementary material for: Parenting and oral health in an inner-city environment: a qualitative pilot study
Source: BMC Oral Health. 2018 Oct 20;18:168. doi: 10.1186/s12903-018-0584-5 (PMC6196005; doi:10.1186/s12903-018-0584-5)
Supplement: Supplementary file 1 — Appendix 1: Topic guide for focus groups and individual interviews. (DOCX 138 kb) [file 12903_2018_584_MOESM1_ESM.docx]

**Topic guide for focus groups and individual interviews**

**Introduction and Welcome**

Outline study aims and methods

Provide opportunity for potential participants to ask questions

Obtain written consent for those wishing to participate in the study

**Oral health**

1. Explore where they get your oral health information from; where do their children get their oral health information from?
2. Explore views on children’s’ teeth? Explore what parents/carers are doing to support their child’s dental health (teeth and gums) *including:*

- Toothbrushing
- Use of fluoride (toothpaste, mouthwash, varnish)
- Diet (food and drink)
- Medicines (sugar-based)

**Attitudes to dental care delivery systems**

1. Has your child/children ever attended a dentist?

If YES, tell us about your experiences of taking your family to visit a dentist?

- explore good experiences and
- bad experiences.

1. Explore the barriers to using dental services including

- Fear
- Cost
- Perceived need
- Features of the dentist
- Features of the environment
- Other: time

1. Explore what would help families to have a positive visit to the dentist including

- language,
- waiting areas,
- quality of dental care,
- relationships with dental team,
- information/communication,
- prevention,
- opening times,
- family friendly features,
- costs

1. Explore how and where might families with young children be able to hear about dental services?

Explore: Word of mouth, Social centres, GPs, Dentist, Children’s centres, schools, local newspapers (which) after school programmes…..

**Community Initiatives**

1. How do you feel about having community initiatives, and would you allow your child to take part, why?

- Dental health education
- Toothpaste and brushing
- Fluoride varnish
- Other

1. Where best delivered and by whom?

Explore what public health initiatives could support them in helping them look after their family’s teeth. Explore the following:

- by dental nurses, dentists, students
- in practice, nursery/school, mobile dental clinics

**
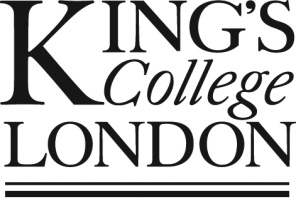
**

**Participant Information Sheet**

*RESC ref no:* **BDM/13/14-29**

**Study Title: Parental/Guardians’ views on Oral Health Promotion initiatives and access to dental care**

1. **Do you live on or near to the Vauxhall gardens Estate?**

- Yes, on the Estate
- Yes, near to the Estate
- No

1. **If you answered yes to Question 2 can you tell us how long have you lived in the area?**

________________ years

1. **Are you?**

- Male
- Female

1. **Which of the following best describes your ethnic group?**

- White
- Black/African/Caribbean/Black British
- Mixed/Multiple ethnic groups
- Asian/Asian British
- Other ethnic group, please state________________________________

1. **What age group do you fall into?**

- 18- 24
- 25 -34
- 35 -44
- 45-54
- 55-64
- 65+

1. **How many children live with you in your home?** _______________________________
2. **Please can you tell us their ages?**

**Child 1: years**

**Child 2: years**

**Child 3: years**

**Child 4: years**

**Child 5: years**

**Thank you**
