# Supplementary material for: Parenting and oral health in an inner-city environment: a qualitative pilot study
Source: BMC Oral Health. 2018 Oct 20;18:168. doi: 10.1186/s12903-018-0584-5 (PMC6196005; doi:10.1186/s12903-018-0584-5)
Supplement: Supplementary file 2 — Appendix 2: Questionnaire for participant demographic data (‘Participant information Sheet’). (DOCX 107 kb) [file 12903_2018_584_MOESM2_ESM.docx]

**
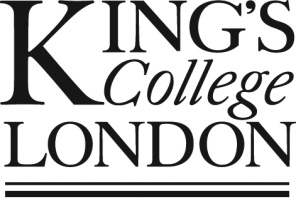
**

**Participant Information Sheet**

*RESC ref no:* **BDM/13/14-29**

**Study Title: Parental/Guardians’ views on Oral Health Promotion initiatives and access to dental care**

1. **Do you live on or near to the Vauxhall gardens Estate?**

- Yes, on the Estate
- Yes, near to the Estate
- No

1. **If you answered yes to Question 2 can you tell us how long have you lived in the area?**

________________ years

1. **Are you?**

- Male
- Female

1. **Which of the following best describes your ethnic group?**

- White
- Black/African/Caribbean/Black British
- Mixed/Multiple ethnic groups
- Asian/Asian British
- Other ethnic group, please state________________________________

1. **What age group do you fall into?**

- 18- 24
- 25 -34
- 35 -44
- 45-54
- 55-64
- 65+

1. **How many children live with you in your home?** _______________________________
2. **Please can you tell us their ages?**

**Child 1: years**

**Child 2: years**

**Child 3: years**

**Child 4: years**

**Child 5: years**

**Thank you**
